# Can You Sequence Ecology? Metagenomics of Adaptive Diversification

**DOI:** 10.1371/journal.pbio.1001487

**Published:** 2013-02-19

**Authors:** Christopher J. Marx

**Affiliations:** 1 Department of Organismic and Evolutionary Biology, Harvard University, Cambridge, Massachusetts, United States of America; 2 Faculty of Arts and Sciences Center for Systems Biology, Harvard University, Cambridge, Massachusetts, United States of America

## Abstract

Few areas of science have benefited more from the expansion in sequencing capability than the study of microbial communities. Can sequence data, besides providing hypotheses of the functions the members possess, detect the evolutionary and ecological processes that are occurring? For example, can we determine if a species is adapting to one niche, or if it is diversifying into multiple specialists that inhabit distinct niches? Fortunately, adaptation of populations in the laboratory can serve as a model to test our ability to make such inferences about evolution and ecology from sequencing. Even adaptation to a single niche can give rise to complex temporal dynamics due to the transient presence of multiple competing lineages. If there are multiple niches, this complexity is augmented by segmentation of the population into multiple specialists that can each continue to evolve within their own niche. For a known example of parallel diversification that occurred in the laboratory, sequencing data gave surprisingly few obvious, unambiguous signs of the ecological complexity present. Whereas experimental systems are open to direct experimentation to test hypotheses of selection or ecological interaction, the difficulty in “seeing ecology” from sequencing for even such a simple system suggests translation to communities like the human microbiome will be quite challenging. This will require both improved empirical methods to enhance the depth and time resolution for the relevant polymorphisms and novel statistical approaches to rigorously examine time-series data for signs of various evolutionary and ecological phenomena within and between species.

## Abstract 2

Identifying evolutionary and ecological phenomena directly from sequencing of microbial communities is surprisingly challenging, even for one as simple as a single species adapting and diversifying in the laboratory.

## Introduction

The capacity of current sequencing technologies has revolutionized fields such as microbial ecology and evolution. Research projects and entire careers have been invented. For example, it has now become respectable, indeed fashionable, to sequence poop. Mouse poop, human poop: it is officially a cottage industry. Why? The microbial flora that outnumber our cells 10-fold and have a total gene content 100-fold greater than our own genome are finally getting the credit (or blame) they deserve for the diverse ways in which they affect our health.

But how much can be gleaned from sequencing alone? The direct sequencing of mixed communities (i.e., metagenomics) and subsequent annotation generates fantastic hypotheses of the functions various members are engaged in. From the perspective of population biology, it is thrilling to know that somewhere in the petabytes of data are the mutations that underlie processes such as evolutionary adaptation or ecological interactions. But which ones? For example, which signals are present in time-course data that could distinguish typical adaptation of a microbe to a single niche from whether it had also diversified into multiple specialists occupying distinct niches? Given the tremendous layers of complexity in our gut community, the challenge is formidable.

## Experimental Evolution as a Model Approach to Understand Natural Communities

Analogous to how classical model systems like *Escherichia coli* and its phage helped unlock the basics of molecular biology, the same sorts of systems have been used to understand fundamental evolutionary processes during adaptation in the laboratory [1]. Most work has been necessarily phenomenological; the genetic basis of adaptation was nearly impossible to uncover prior to genome resequencing. A senior colleague of mine once quipped (and I have previously relayed [2]) that experimental evolution was “population genetics without the genetics.” Times have changed. As with the poop-omics described above, researchers can now sequence isolates [3],[4] or mixed samples [5] of evolving populations, thereby uncovering the mutations that occur, as well as changes in their frequencies over time.

**Figure 1:**
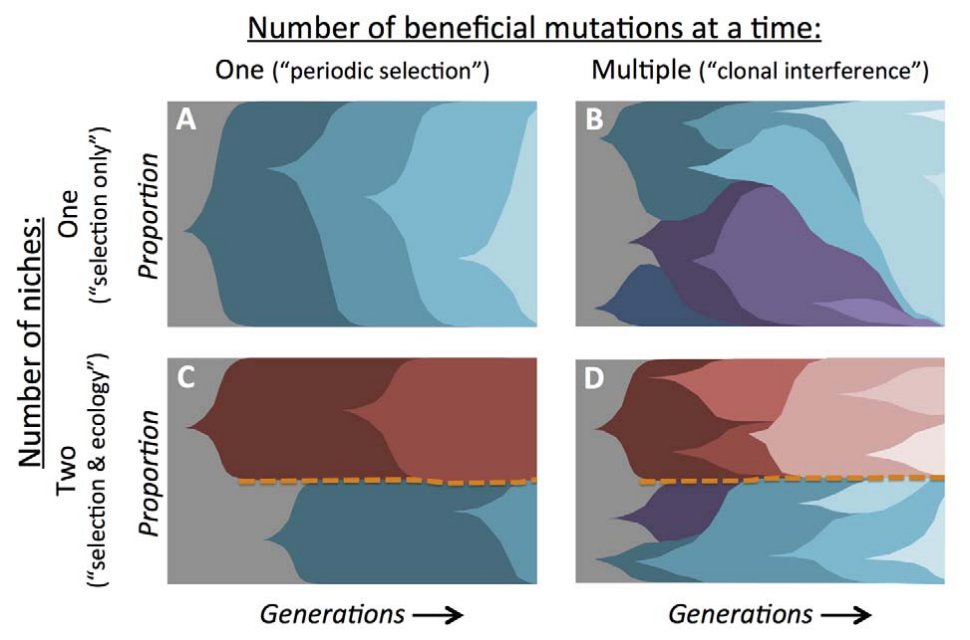
Dynamics of allele frequencies under different evolutionary and ecological scenarios. These diagrams indicate the proportion of alleles through time, with each color series representing those that arose from a common first mutation upon the ancestral (gray) genotype. A) The canonical model for adaptation in a single niche has been one of periodic selection, whereby beneficial mutations occur rarely enough that only one ever rises through the population at a time. B) Experimental evolution has repeatedly shown that many beneficial mutations can occur simultaneously and compete with each other before any one of them fixes, a scenario known as clonal interference. C) If multiple ecological niches exist, selection can drive a lineage to split into multiple, coexisting phenotypes (i.e., adaptive diversification). Lineages in each niche are indicated by either warm or cool colors and are separated by an orange dashed line representing the apparent equilibrium. Fixation events occur within each niche without eliminating diversity in the other niche. D) Both clonal interference and ecological diversification can operate simultaneously, giving rise to multiple lineages competing within each niche.

Which patterns should be expected from population sequencing in the simplest imaginable scenario: one (asexual) genotype of one species grown on one nutrient in a closed system (without migration)? If I had taken population genetics, I would have been told the gospel that past selection has already rendered most organisms to near perfection, thus almost all new mutations are neutral or deleterious. Beneficial ones are so incredibly rare (and mainly of small effect) that populations would have to wait a substantial time for something good enough to come along and escape random loss. Once established, however, that new rock-star genotype could rise to fixation (perhaps with other, more-or-less neutral mutations that could hitchhike with it), unchallenged as it outcompetes the homogenous sea of unimproved genotypes around it. The mutated genotype would become the new normal, destined to linger until the process repeats itself. This idealized model of steplike improvements is termed “periodic selection” and, until recently, formed the basis of much of evolutionary theory regarding adaptation [6]. Furthermore, depending upon how many ways a given genotype might improve, replicate populations may fix mutations in parallel functions, genes, or even nucleotides. Indeed, parallelism has been quite commonly observed in evolution experiments [7]–[12]. Periodic selection would give an extremely clear metagenomic signal: rarely a single new allele would rise in frequency exponentially through time, and after a while, a second one (on the background of the first, Figure 1A). It is a shame that reality does not live up to this ideal.

A first complication to periodic selection arises because typical experimental populations have been sufficiently large to have multiple beneficial mutations arise and vie for fixation simultaneously (Figure 1B). Just like when several new companies dive into a market at the same time, your business model has to be both viable and better than those of all of your competitors. Amongst asexual organisms this is known as “clonal interference” [13], and it biases winning mutations toward those with the largest selective effects likely to occur at that population size. Clonal interference also drags out fixation events, providing time for further beneficial variants to arise from competitors before any of them have fixed [14]. This will wreak havoc on metagenomic data. Although there will still be rapid changes in allele frequencies as expected for periodic selection, now there will be many lineages transiently rising and falling as they continue to mutate and compete. There is growing evidence from multiple approaches for exactly these sorts of dynamics [5] [15]–[18].

The second major complication, even in the simple regime of well-mixed environments seeded with a single genotype, is that the ancestral strain can diversify into multiple coexisting ecological specialists. First, this will mean that although some mutations may be generally beneficial, others will only be useful in certain niches. These may, however, occur repeatedly across replicate populations that diversify. Second, selection can drive a lineage to split into multiple specialists in what is called “adaptive diversification” [19]. This can occur when selection becomes “disruptive”, rewarding divergent phenotypes whose fitness is not absolute, but depends upon the frequency of both types. If this “frequency-dependent” selection is both negative (more fit when rare) and has regimes where either type is the best due to trade-offs, this generates a stable equilibrium between the genotypes. Over the long term, this may result in maintenance of multiple lineages that can each continue to adapt to their niche without eliminating lineages in the other niche(s) [20]. Diversification generates rather complicated metagenomic signatures (Figure 1C), all the more so given clonal interference would also be occurring (Figure 1D). The defining difference is whether alleles sweep through the whole species or only appear to affect some of the species' lineages. If sequence data cannot distinguish “simple” competition in one niche from adaptive diversification for *E. coli* in a flask, what are our chances of understanding evolution and ecology in a gut from sequencing what comes out of it?

## Looking in Sequence Data for Signs of Ecological Diversification when You Know It Happened

In this issue of *PLOS Biology*, Herron and Doebeli [21] report metagenomic sequencing from 1,200 generations of adaptation and ecological specialization of *E. coli* in the laboratory. One of the key advantages of this study is the backdrop of a rich history of earlier papers that characterized parallel diversification across replicate populations that evolved in a mixture of glucose and acetate [22]–[25]. Their ancestral strain grows quickly on glucose, and then slowly switches to eating the much less desirable acetate. In each of the ten populations evolved on glucose and acetate, two distinct evolved phenotypes emerged: one that grows even more rapidly on glucose but takes longer to transition to acetate (slow-switchers, SS), and another that is not as fast on glucose as SS but can immediately adjust to grow on acetate (fast-switchers, FS) [24]. Either phenotype can invade the other when rare, coming to a stable equilibrium [22]. Furthermore, both the likelihood of FS emerging [24] and the benefit of particular mutations within this lineage [25] have been shown to depend upon whether the SS phenotype had already evolved. Some of the genetic basis of these phenotypes had been worked out previously [23],[25], and this paper extends these analyses by first sequencing a dozen isolates representing known SS or FS phenotypes from three populations. The major data, however, was metagenomic sequencing of time series to test whether raw sequence data could capture that adaptive diversification took place.

Parallel beneficial mutations already gave some hint of adaptation to multiple ecological strategies. While a few genes were targets for beneficial mutations across populations and strategies (distinct deletions of the ribose operon, Δ*rbs*, in all but one lineage), others seemed to be specific to each niche. In all three populations, the SS phenotype started with Δ*rbs* and mutations in *spoT*, a global regulator of the transition from growth and starvation. The next mutation in the SS lineages was nearly always in *nadR*, which encodes a multifunctional enzyme/regulator of NAD biosynthesis. On the other hand, the FS phenotype always started with changes in acetate metabolism (mutations in one or more of *ackA*, *pta*, or *ptsG*). The repeated observation of the same *pair* of mutational patterns is consistent with the presence of two ways to improve in all replicate populations.

The temporal dynamics of allele frequencies showed many complicated rises and falls, a few of which clearly indicated ecological interactions. There were multiple lineages, reversals in the direction of allele-frequency changes, and no fixations over 1,200 generations; all of these are qualitatively indistinguishable from previous observations of clonal interference in single-resource environments [5] [15]–[18]. The major signal of ecological diversification, however, came when genotypes rose in frequency to exclude some lineages, but then stabilized with respect to others that appeared to be “immune” to their advantage (like Figure 1C–D). This is a clear violation of transitivity for fitness expected in a single-niche environment, and thus indicates some sort of diversification into multiple niches.

One utility of sequencing is to unveil the evolved alleles that likely caused specialization and the resulting coexistence. A great advantage of laboratory experiments is the ease of directly testing these hypotheses by reconstructing communities with different genotypic (or species) composition. For example, the authors of the present study suggest *nadR* alleles in the SS lineages were beneficial only after the FS lineage arose. Alternatively, since the *nadR* alleles consistently rose after the Δ*rbs* and *spoT* mutations occurred in their own lineage, perhaps their benefit was modified by earlier mutations in their lineage, as has been found in other studies [17],[26],[27] including one of the authors' own [25]. So did *nadR* alleles arise because of between-organism coevolution, within-genome epistasis, both of these effects, or neither of them? Thankfully, these sorts of questions can be answered definitively in resynthesized communities.

## Implications for Natural Communities and Future Challenges

For communities that can be observed but not easily manipulated—such as the human gut—can sequencing alone identify adaptation of its members or ecological interactions between them? Despite known adaptive diversification, it should be noted that surprisingly little of the temporal dynamics of the two-niche *E. coli* population unambiguously defy what is possible from simple selection. But are there further, more nuanced aspects of time-series data such as these that would not jibe with simple selection in a single niche? On the empirical side, such quantitative analyses would benefit tremendously from more precise data (more reads per timepoint for the polymorphisms in question) and greater temporal resolution of populations. For example, my laboratory recently developed FREQ-Seq, which barcodes samples and eliminates library preparation in a manner that can generate ∼10^5^ reads per allele per timepoint for thousands of timepoints in a single Illumina lane [28]. On the computational front, there is a clear need for statistical models that can rigorously interpret the temporal dynamics for signs of selection and/or niche differentiation between genotypes of individual species within sequenced communities. These within-species analyses can then be integrated with methods that infer ecological dynamics between species from their correlated abundances [29].

A final fascinating, and somewhat sobering, lesson from Herron and Doebeli is that one species can rapidly evolve to behave like two due to just one or two mutations. Consider the converse situation: that multispecies communities sometimes have been characterized as a much smaller number of “guilds,” comprised of species with relatively similar niches [30]. Collectively, these two concepts would generate a quite fluid scenario whereby one species can quickly act like several; and many already present may act like one. This potential blurring of ecology and evolution implies that beneficial mutations in one species could drive an unrelated species (with a similar niche) to extinction, while sparing extremely closely-related, recently diverged genotypes of its own species. And if this was not headache enough, throw in horizontal gene transfer, which has been inferred to be particularly common in environments such as the gut [31]. It is clear that studies of microbial evolution and ecology in natural communities will remain challenging and interesting for a long time. It is equally clear that systems as simple as “just *E. coli* in a flask” have many lessons left to teach us.
